# Effect of biomass immobilization and reduced graphene oxide on the microbial community changes and nitrogen removal at low temperatures

**DOI:** 10.1038/s41598-020-80747-7

**Published:** 2021-01-12

**Authors:** Anna Banach-Wiśniewska, Mariusz Tomaszewski, Mohamed S. Hellal, Aleksandra Ziembińska-Buczyńska

**Affiliations:** 1 Environmental Biotechnology Department, Faculty of Power and Environmental Engineering, Silesian University of Technology, Akademicka 2, 44-100 Gliwice, Poland; 2 Water Pollution Research Department, National Research Centre, 33 El-Behooth St., Dokki, Cairo, Egypt

**Keywords:** Biotechnology, Molecular biology, Environmental sciences

## Abstract

The slow growth rate and high optimal temperatures for the anaerobic ammonium oxidation (anammox) bacteria are significant limitations of the anammox processes application in the treatment of mainstream of wastewater entering wastewater treatment plant (WWTP). In this study, we investigate the nitrogen removal and microbial community changes in sodium alginate (SA) and sodium alginate–reduced graphene oxide (SA-RGO) carriers, depending on the process temperature, with a particular emphasis on the temperature close to the mainstream of wastewater entering the WWTP. The RGO addition to the SA matrix causes suppression of the beads swelling, which intern modifies the mechanical properties of the gel beads. The effect of the temperature drop on the nitrogen removal rate was reduced for biomass entrapped in SA and SA-RGO gel beads in comparison to non-immobilized biomass, this suggests a ‘‘protective” effect caused by immobilization. However, analyses performed using next-generation sequencing (NGS) and qPCR revealed that the microbial community composition and relative gene abundance changed significantly, after the implementation of the new process conditions. The microbial community inside the gel beads was completely remodelled, in comparison with inoculum, and denitrification contributed to the nitrogen transformation inside the beads.

## Introduction

Biological wastewater treatment is an important biotechnological application with microorganisms being crucial to its success. Nitrogen plays a significant role amongst the compounds that should be completely removed from wastewater due to the contribution of eutrophication of the receiving waters, ammonia toxicity, and direct effect on aquatic life. Nitrogen removal from wastewater comprises of three main processes: denitrification, nitrification, and anammox (anaerobic ammonium oxidation)^1,2^. Over the past few decades, the anammox process is gaining more and more interest and is considered a cost-effective and eco-friendly method. Process is carried out by chemolithoautotrophic bacteria belonging to *Planctomycetes,* which are capable to oxidize ammonium under anoxic conditions using nitrite as the electron acceptor^3,4^. The anammox application used in the mainstream of a municipal wastewater treatment plant (WWTP) could offer a significant reduction of operating costs and greenhouse gas emission. Despite several advantages over denitrification and nitrification, the anammox application still has its limitations. The optimal temperature for anammox bacteria utilisation in these biotechnological process is 35 ± 3 °C, which is a limitation for anammox process applications in the mainstream of a municipal WWTP as the average temperature is close to 15 °C^5,6^. Moreover, the anammox biomass has a slow growth rate which causes significant problems with washing out the anammox bacteria from the systems^7–9^. Considering these factors, finding a way to maintain the biomass in the reactors and to perform an effective nitrogen removal process at low temperature would open the possibilities of a wide-ranging extended anammox process application.

Numerous studies have reported the improvement of the anammox process efficiency by the nanoparticles addition. They include nickel (II) oxide, which increased the nitrogen removal rate by 9% in concentration of 5 mg/L^10^, maghemite (γ-Fe_2_O_3_)—stimulated activity by 43% after 200 mg/L addition^11^ and zero valent iron (ZVI)—increased anammox activity by 58% with concentration in rage from 0.0004 to 0.004 mg/L^12^. Our previous work has shown that the activity of anammox bacteria at temperatures below 20 °C (called “cold anammox”) can be enhanced by reduced graphene oxide (RGO) addition^13,14^. It was noted that the increase in the anammox activity tends to be stronger at temperatures lower than 20 °C and the maximum stimulation of the anammox bacteria activity was observed at 13 °C. During short- and long-term experiments 28% and 17% stimulation of the anammox process was observed, respectively. The support of the anammox process via RGO addition was the aim of research by Yin et al., where fast start-up of the process was achieved^15^ and the activity of anammox biomass and key enzymes was stimulated^16^.

Despite promising improvements in cold anammox stimulation by using nitrogen removal supported nanomaterials such as RGO, it still does not solve the problems with fixing both the biomass and the nanoparticles in the reactor. Thus, it is important to find the way, which allows supporting the nitrogen removal process at low temperatures, protects the microorganisms from changeable environmental conditions and will help to retain the nitrogen cycle microorganisms in the treatment system.

In the last decade, activated sludge immobilization techniques have been widely considered useful for wastewater treatment^17–19^. One method for biomass immobilization which is gaining recent interest is a gel entrapment as it has the ability to increase the stability of the process and tolerance of microorganisms in changeable environmental conditions^20,21^. Gel entrapment helps to maintain the biomass in the reactor which is crucial for slow-growing nitrogen removal bacteria such as anammox bacteria.

The aim of this study is to combine the biomass immobilization with RGO addition to increase nitrogen removal efficiency at low temperature. Herein two carriers: sodium alginate (SA) and sodium alginate–reduced graphene oxide (SA-RGO) gel beads were prepared using a cross-linking method. SA is a natural polysaccharide composed of (1,4)-linked β‑d‑mannuronate (M) and α‑l‑guluronate (G) residues and is considered as a suitable polymeric matrix due to its high stability in organic solvents, the ease in which it undergoes immobilization via cross-linking in the presence of multivalent cations, and its nontoxicity^22^. However, compared with synthetic polymers, SA exhibits several disadvantages, such as strong hydrophilic character, poor mechanical resistance, and low thermal stability^23–25^. An effective approach for improving the SA properties for wastewater treatment is to fill nanoparticles into the SA matrix^26–28^.

Considering the described limitations of the anammox process, its application, and the properties of SA and RGO, the nitrogen removal efficiency was monitored in the SA and SA-RGO carriers, considering the dependence of temperature, and with a special emphasis on the temperature close to the mainstream of the WWTP: 15 ± 3 °C.

When considering the nitrogen removal it is important to determine the microbial diversity and the dynamics characteristics. There is much research studying the process performed by particular species entrapped in gel beds, such as anammox bacteria, denitrifying bacteria, and nitrifying bacteria^24,29,30^. However, only a handful have investigated the diversity and relative abundance of the whole activated sludge microbial community entrapped within the gel beads^31–34^. In technological systems activated sludge is the heterogeneous community of microorganisms of different species. The exploration of microbial diversity and relationships between bacterial groups are crucial for process optimization and effective wastewater treatment. This is why in this study, in addition to process efficiency, we are focusing on the proportion of particular bacterial groups and changes in the microbial community structure in relation to the temperature, immobilization methods, and the RGO addition.

## Materials and methods

### Biomass characteristics

Biomass was taken from laboratory scale anammox sequencing batch reactor (SBR; a volume of 5 L), where it was acclimated for 6 months. The SBR was previously inoculated with anammox activated sludge from full-scale deammonification SBR situated in Germany. The reactor operated in stable conditions at a temperature of 30 ± 1 °C and at a pH of 7.5 ± 2. The biomass was fed with simulate sewage medium with the following composition: 0.725 g NH_4_Cl L^−1^, 1.268 g NaNO_2_ L^−1^, 0.048 g KHCO_3_ L^−1^, 0.041 g KH_2_PO_4_ L^−1^, 0.228 g MgSO_4_ 7H2O L^−1^, 0.007 g FeSO_4_ 7H_2_O L^−1^, and 0.004 g EDTA L^−1^. The biomass composition was revealed via next-generation sequencing (NGS) and inoculum which consisted mostly of Ca*. Brocadia* (30.57%), unclassified bacteria (27.6%), *Dokdonella* (7.72%), Ca*. Scalindua* (3.96%), and *Nitrospira* (2.17%).

### Preparation of the carriers

SA gel beds were prepared by dropping the 4% SA (purchased from Sigma-Aldrich) solution into a crosslink solution (2% calcium chloride). In turn, to obtain combined RGO-SA beds, 4 g of SA was added into 100 mL RGO solution (0.28 g*L^−1^). Reduced graphene oxide is commercially available (Nano Carbon Sp. z o.o., Poland) and had the following properties: a bulk density of the dry powder: 0.15–0.25 g*mL^−1^, elemental analysis (percentage by weight): C > 85%, H < 1%, N < 3.5%, O < 10%, other elements ~ 0.6%, and X-ray fluorescence (XRF) impurities analysis: Cl (0.3%), Mn (0.2%), S (0.01%), K (0.01%), Fe (0.01%), Ca (0.009%), Cu (0.006%), and Ni (0.001%). SA-RGO solution was stirred vigorously for 12 h then the concentrated anammox biomass was added into the SA-RGO at a ratio of 2:1 (v/v). The mixture was dropped through a peristaltic pump into a 100 mL of coagulation solution containing CaCl_2_ (2% w/v) under continuous stirring. Bacteria were immobilized for 1 h. Subsequently, the obtained pellets were washed with deionized water. The obtained beads were ready to use after a reactive period of 12 h to limit the effect of the immobilized process to the biomass.

### Carrier characterisation

#### Scanning electron microscopy

The morphology and structure of the control and immobilized samples were characterized using scanning electron microscopy (SEM, QUANTA FEG 250, Thermo Fisher). The samples were washed with a phosphate buffer saline solution and then were dried thoroughly at room temperature with a silica gel dryer. Next, the samples were sprayed with gold and were covered using a vacuum cover system for the evaporation of metals.

#### Water-related properties

Each water-related parameter was measured on plain gel beads (without biomass) with 10 replications. To perform the water swelling assessment, the carriers were initially weighed, then immersed in distilled water (analytical balance, OHAUS, accuracy = 0.0001 g). The samples were taken out every 1 min, wiped carefully with filter paper, and weighed again. The experiment was continued until there was no further absorption of water. The results presented show the change in bead weight during the swelling as a percentage of the swelling ratio (%SR).

To calculate the water retention capacity (%WRC), the SA and SA-RGO beds were weighed. Next, the beads were dried in an oven at 105 °C overnight and weighed again. The WRC was expressed as a percentage ratio of the water contained in the sample before drying relative to its dry weight, and was calculated using the formula (1):

1 $$ {\% }{\text{WRC}} = \frac {{\text{W}}_{\text{never-dried}}-{\text{W}}_{\text{dry}}}{{\text{W}}_{\text{dry}}} {100 \%}$$

where W_never-dried_ is the weight of the SA and SA-RGO before drying and W_dry_ is the dry weight of the sample.

To calculate the shrinking factor (SF), the diameter of the swollen (d_wet_) and the diameter of completely dried, in an oven at 105 °C overnight, (d_dry_) beads were measured and the SF was calculated according to the following formula (2):

2 $$ SF = \frac{{\left( {d_{wet} - d_{dry} } \right)}}{d_{wet} } $$

In the water holding study, the SA and SA-RGO beads were immersed in distilled water to obtain a maximum absorption level, wiped carefully with filter paper, placed on an analytical balance, weighed, incubated for 48 h at ambient temperature (25 °C), and weighed again. During the incubation the water lost was measured and monitored over time in the moisture analyser (AXIS, accuracy = 0.0001 g) to obtain W_dwet_ parameter. The water holding capacity (%WHC) was calculated using the following formula (3):

3 $$ {\% }{\text{WHC}} = \frac {{\text{W}}_{\text{wet}}-{\text{W}}_{\text{dwer}}}{{\text{W}}_{\text{dry}}} {100 \%}$$

where W_wet_ is the weight of the swollen beads, W_dwet_ is the weight of the swollen beads during drying, and W_dry_ is the dry weight of the sample^35^.

#### Equilibrium absorption capacity

SA and SA-RGO beads were first put into distilled water until equilibrium swelling states were achieved. Methylene blue (MB) was used to test the absorption capacities of the pellets. Then equilibrium swollen hydrogels were added into the MB aqueous solutions, and dye absorption experiments were performed at 15 °C, 23 °C and 30 °C. Measurement of the changes in the MB concentration over time allowed one to estimate the absorption capacity of the SA and the SA-RGO carriers. Samples of upper layer liquid were collected during the experiment at regular intervals, then the absorption was measured at a wavelength of 665 nm using a UV–VIS spectrophotometer. The removal rate (R_MB_) and the equilibrium absorption capacity coefficient (q_MB_) were calculated as follows (4, 5):

4 $$R_{MB} = \frac{{{ }C_{0} - C_{e} }}{{C_{0} }} \times 100\%$$

5 $$q_{MB} = \frac{{\left( {{ }C_{0} - C_{e} } \right) \times V}}{m}$$

where C_0_ (mg L^−1^) and C_e_ (mg L^−1^) are the initial and equilibrium concentrations of the MB solutions, respectively, m (g) is the weight of the pellets, and V (L) is the volume of the MB solution^36,37^.

### Experimental setup and analytical methods

**Figure 1:**
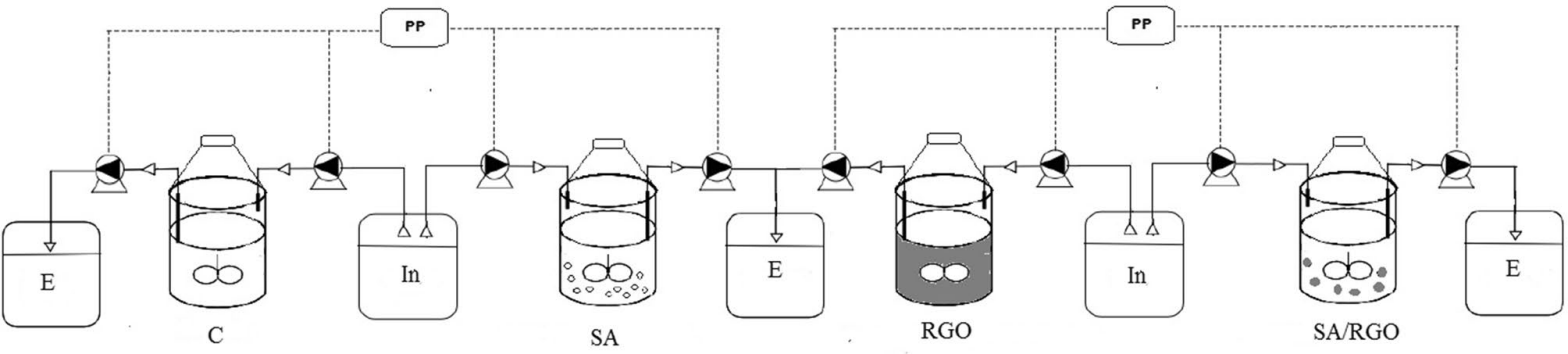
The scheme of the SBRs used in the experiment: *C* control, *SA* biomass immobilized in SA, *RGO* biomass with RGO addition, *SA-RGO *biomass immobilized in SA-RGO gel beads, *PP* peristaltic pump, *E *effluent, *In* Influent.

Three methods based on gel entrapment and RGO addition were used to support the biological nitrogen removal. The experiment was conducted in four SBRs, with a volume of 1 L, and the process was performed at 23 °C and 15 °C (Fig. 1).

The SBRs were inoculated with an equal amount of biomass and VSS (1.432 ± 0.634 g*L^−1^). During the experiment, the reactors were operated at a pH level of 7.5 ± 0.4 and the pH was corrected using 10% HCl and 10% NaOH. The dissolved oxygen (DO) concentration was below 0.2 mg*L^−1^, and the hydraulic retention time (HRT) was equal to 2 days. The reactor was fed with a mineral medium containing: 0.048 g KHCO_3_ L^−1^, 0.041 g KH_2_PO_4_ L^−1^, 0.228 g MgSO_4_ 7H_2_O L^−1^, 0.007 g FeSO_4_ 7H_2_O L^−1^, and 0.004 g EDTA L^−1^. The nitrogen concentration was regulated using both NH_4_Cl L^−1^ and NaNO_2_ L^−1^.

The concentrations of the N-NH_4_, N-NO_2_ and N-NO_3_ were measured by photometric tests (MERCK Milipore) with a photometer (MERK Spectroquant NOVA 60). The concentrations of the VSS were measured using a standard method^38^.

### Molecular biology analyses

#### Sampling and DNA isolation

The samples were collected from inoculum and each SBR at each of the tested temperatures. Then, samples were preserved with RNAlater (Promega) to prevent DNA degradation. Before the DNA isolation the samples were washed with 1 × phosphate buffer saline in order to remove potential PCR inhibitors: samples with buffer addition were stirred vigorously, centrifuged (1 min, 1500 rpm), then the supernatant was removed. The procedure was repeated thrice. The samples were stored at − 40 °C until DNA isolation. The DNA was isolated using a mechanical method described previously by Ziembińska-Buczyńska et al.^39^. Isolated DNA was treated with an Antyinhibitor Kit (A&A Biotechnology) and purified with a Clean up Kit (A&A Biotechnology) according to manufacturer instruction.

#### qPCR

qPCR analysis was completed based on the functional genes, specific for anammox bacteria, denitrifiers, NOB and AOB (Table 1). Details for the qPCR procedure and sequence of the primers are described elsewhere^39,40^. All reactions were conducted at least in triplicates. The relative genes abundancy (q) was calculated using the following formula (6):

6 $$q = 2^{{\Delta C_{t} }}$$

where Ct is C_t_ the value for the analysed gene and Δct = C_tref_ − C_tanal_, C_tref_ is the C_t_ of the bacterial 16S rRNA gene (used as a reference gene).

**Table 1:** Functional genes used in the qPCR analysis.

Specifity	Target gene	Encoding enzyme	References
Bacteria	16S rRNA	Universal bacterial marker gene used as reference gene	^69^
Ammonia oxidizers	*Amo*	Ammonia monooxygenase	^60^
Nitrite oxidizers	*Nrx*	Nitrite oxidoreductase	^70^
All known *Planctomycetes*	*Hzo*	Hydrazine oxidoreductase	^71^
Denitrifiers	*NirS*	Nitrite reductase	
	*NirK*		

### Next-generation sequencing

Amplification of the 16S rRNA coding gene fragment was performed with S-D-Bact-0341-b-S-17 (5′ TCGTCGGCAGCGTCAGATGTGTATAAGAGACAGCCTACGGGNGGCWGCAG 3′) and S-D-Bact-0785-a-A-2: (5′ GTCTCGTGGGCTCGGAGATGTGTATAAGAGACAGGACTACHVGGGTATCTAATC3 ′) primers^41^ and the NEBNext High-Fidelity 2 × PCR Master Mix according to the manufacturer’s protocols (Bio Labs Inc., USA). The samples were dual indexed using the Nextera XT Index Kit. The sequencing procedure was performed with the MiSeq sequencer by applying paired-end technology, 2 × 250 nt with a MiSeq Reagent Kit V2 (Illumina, USA) according to the manufacturer’s instructions. The obtained data were automatically analysed using a MiSeq Reporter (MSR) v2.4 software (Illumina, USA). MSR was used in order to de-multiplex the raw data and to analyse each sample down to the species level. The analysis started with de-multiplexing filtered indexed reads. Summary files of the percentage hits and rarefaction files were generated by MSR to enable further data analysis. FASTQ files were generated and included information of each of the sample reads and quality scores. The classification step used ClassifyReads, an algorithm that provides a species-level classification for the paired-end reads. The reads classification on each taxonomical level was performed by conducting a comparison with the reference sequences stored in the Greengenes (http://greengenes.lbl.gov v13_5) database. Preparations of the reference database consisted of reads containing more than 50 degenerated nucleotide filtering reads shorter than 1250 base pairs and reads with an unclear taxonomical position^42^.

### Statistical analyses

A Shapiro–Wilk test was performed to test the data normality and a Leven’s test was performed to test the equality of the variances. *T* test, *t* test with Cochran–Cox adjustment, and two-way analysis of variances (ANOVA) were used to examine the significance of the differences between results obtained from the SBRs with different experimental conditions and the water-related properties of the gel beads. The tests were selected based on the relevant data. In addition, Spearman’s rank correlation coefficients were calculated for the relative gene abundancy results. A significance level α = 0.05 was assumed in all the performed statistical analysis.

## Results and discussion

### Carrier characteristics

**Figure 2:**
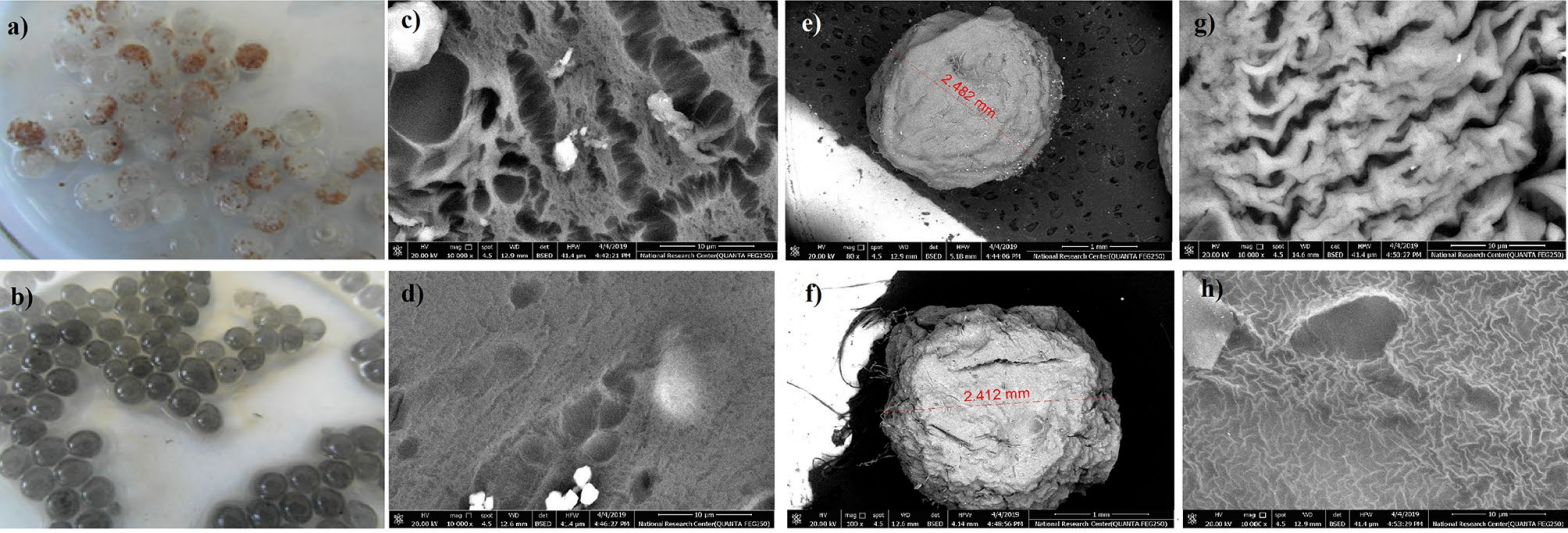
The SA (**a**) and SA-RGO (**b**) gel beads and their corresponding SEM images: surface of SA (**c**) and SA-RGO (**d**) gel beads with immobilized anammox biomass, image of single SA (**e**) and SA-RGO (**f**) bead and the surface of SA (**g**) and SA-RGO (**h**) control gel beads (without immobilized anammox biomass).

To support nitrogen removal at low temperatures, immobilization of SA was combined with RGO addition. Anammox biomass was entrapped in SA and SA-RGO gel beads (Fig. 2a,b). The SEM images provided direct information on the structure and the surface of the beads, as illustrated in Fig. 2c–h. The gel beads surface showed some pores, which indicates that the bead porosity facilitates penetration of nutrients into the inside of beads. Also, the presence of the pleats and pores indicated that some functional groups and adsorption sites exist on the surface. By the addition of RGO to the SA the pores and bulges on the surface decreased (Fig. 2d,h), and the surface of SA-RGO displayed a generally smooth morphology in comparison to the SA beads (Fig. 2c,g).The smooth morphology suggest good dispersion of the RGO nanoparticles in the SA matrix. Although, together with the smoothing of SA-RGO surface, a reduction in pores may be observed. The characteristic pattern on the gel bead surface (Fig. 2h) reflected sheetlike morphology of RGO and may also suggest that RGO tended to cover the surface of the SA, as reduced graphene oxide could form the network structure^43,44^.

Since the gel beads will swell in water, the water-related properties are one of the most important parameters used to evaluate the crosslinking and interactions of the combined gel materials. The diameter of the beads in a swelling equilibrium state were on average 4.8 ± 0.16 mm (SA) and 4.68 ± 0.21 mm (SA-RGO). The reduction in the diameter of the beads in a swelling equilibrium state can be explained by the interfacial interaction between the SA polymer chains and RGO nanoparticles that can restrict the water adsorption towards the SA matrix in aqueous feed solution^45^. Both the SA and SA-RGO beads have high water-holding and retention capacity (Table 2). However, the extent of the water absorption could deform and widen the gel beads as well as weaken the mechanical properties of the pellets. The RGO addition decreased the values of the calculated water-related parameters of SA. Several studies report that suppression of hydrogel swelling may help retain their mechanical properties and their initial shape^22,46,47^. These statistically significant differences were found between the water holding and water retention properties. As reported previously, the filling of the SA matrix with nanoparticles may help to enhance the mechanical properties of the material^15,26,28^. Swelling suppression and well filling of SA matrix with RGO mean that SA-RGO beads have better mechanical properties than pure SA. At the same time, a decrease in the SR, WHC, and WRC of the SA-RGO gel beads does not cause a significant decrease in the absorption capacity, which is expressed as qMB (Table 3). Alginates are biopolymers physically cross-linked by ionic interactions^48^. Thus, it should be pointed that the swelling properties of the gels may be affected by ionic strength and specific ionic comoposition. The RGO addition may have possible impact on increasing the conductivity, while the RGO may act as an excellent conductive agent ensuring electron transfer and ion diffusion^28^. It may be possible, that in the case of SA-RGO, the cross-link density of the gel beads may decrease as a function of decreasing gel beads diameter, what may also has a reflection in described swelling properties. It can be also related to permeating of the biopolymer chains between RGO layers and subsequently cross-linkage of the polymer chains and RGO nanoparticles.

**Table 2:** Water-related properties of the investigated carriers: *SR* swelling ratio, *SF* shrinking factor, *WHC* water holding capacity, and *WRC* water retention capacity. The data is presented as a means value ± standard deviation, α = 0.05.

	SR (%)	SF	WHC (%)	WRC (%)
SA	46.83 ± 9.74	2.67 ± 0.42	217 ± 60.14	4089 ± 448
SA-RGO	28.40 ± 7.38	2.36 ± 0.49	116.76 ± 18.53	3358 ± 250
p-value (*t* test/*t* test with Cochran Cox adjustment)	0.129	0.43	0.006	0.015

**Table 3:** The equilibrium absorption capacity coefficient (q_MB_), methylene blue removal rate (R_MB_), and p values calculated with two-way ANOVA; values p < 0.05 were considered as a statistically significant.

	q_MB_			R_MB_ [%]		
	15 °C	23 °C	30 °C	15 °C	23 °C	30 °C
SA	0.0482	0.0459	0.0447	47.21	31.35	24.68
SA-RGO	0.113	0.0477	0.0467	46.56	33.48	30.72
**p-values (two-way ANOVA)**						
For temperature	p = 0.086			p < 0.001		
For beads type	p = 0.113			p = 0.335		

On the other hand, along with the increase of the useful graphene materials application, questions about their influence on the environment and human health are arising. The minimization risk for human health is required to be taken for consideration in order to develop safe graphene-based technologies. Recently, the opposite opinions presenting biocompatibility or toxicity have been reported, depending on the cells type, the experiment conditions and the specific physiochemical properties of the nanomaterials^49^. The potential toxicity of RGO in biomedical systems is being extensively researched. RGO cytotoxicity can be induced by mechanical cell membrane damage, reactive oxygen species (ROS) generation, inflammation, mitochondrial disorder, apoptosis or necrosis^50^. RGO has been also seen to induce significant increases in both intercellular reactive oxygen species levels and in mRNA levels of thioredoxin reductase and hemeoxygenase 1^51^. The specific effects should be evaluated for the individual applications. Hovewer, concerns remain over the long-term toxicity of RGO and application graphene-based compound in wastewater, especially in the context of human healt and environment.

The water properties and nutrient diffusion are directly connected with the absorption capacity of the materials used for the immobilization. RGO addition to the SA does not cause a significant difference in the absorption capacity (q_MB_) and the methylene blue removal rate (R_MB_). Statistical analysis revealed that the differences in these values are connected with the temperature than the gel bead type. The temperature has a significant impact on the methylene blue removal rate (p < 0.001). Both gels exhibit quite good adsorption capacities of the cationic dye, MB. The highest absorption coefficient is noted in the SA-RGO beads at a temperature of 15 °C.

### Nitrogen removal efficiency

**Figure 3:**
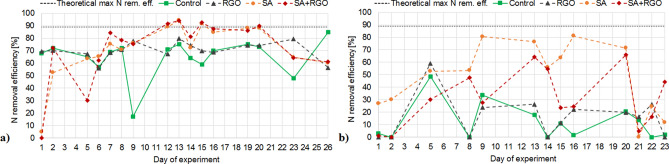
Nitrogen removal efficiency in SBRs operated at (**a**) 23 °C and (**b**) 15 °C.

**Figure 4:**
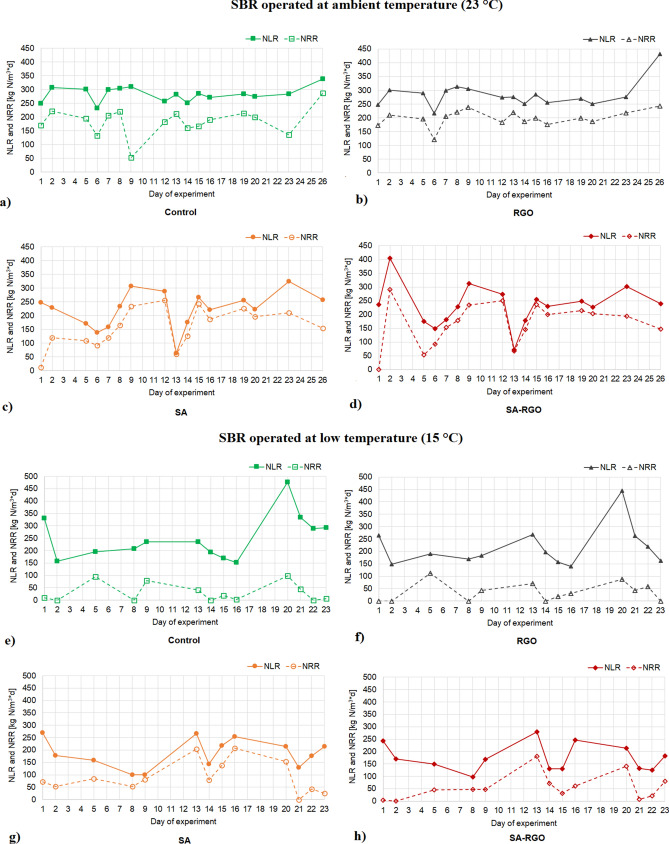
Nitrogen removal rate (NRR) and nitrogen loading rate (NLR) in the SBRs operated at ambient temperature (23 °C) (**a**–**d**) and at low temperature (15 °C) (**e**–**h**).

**Figure 5:**
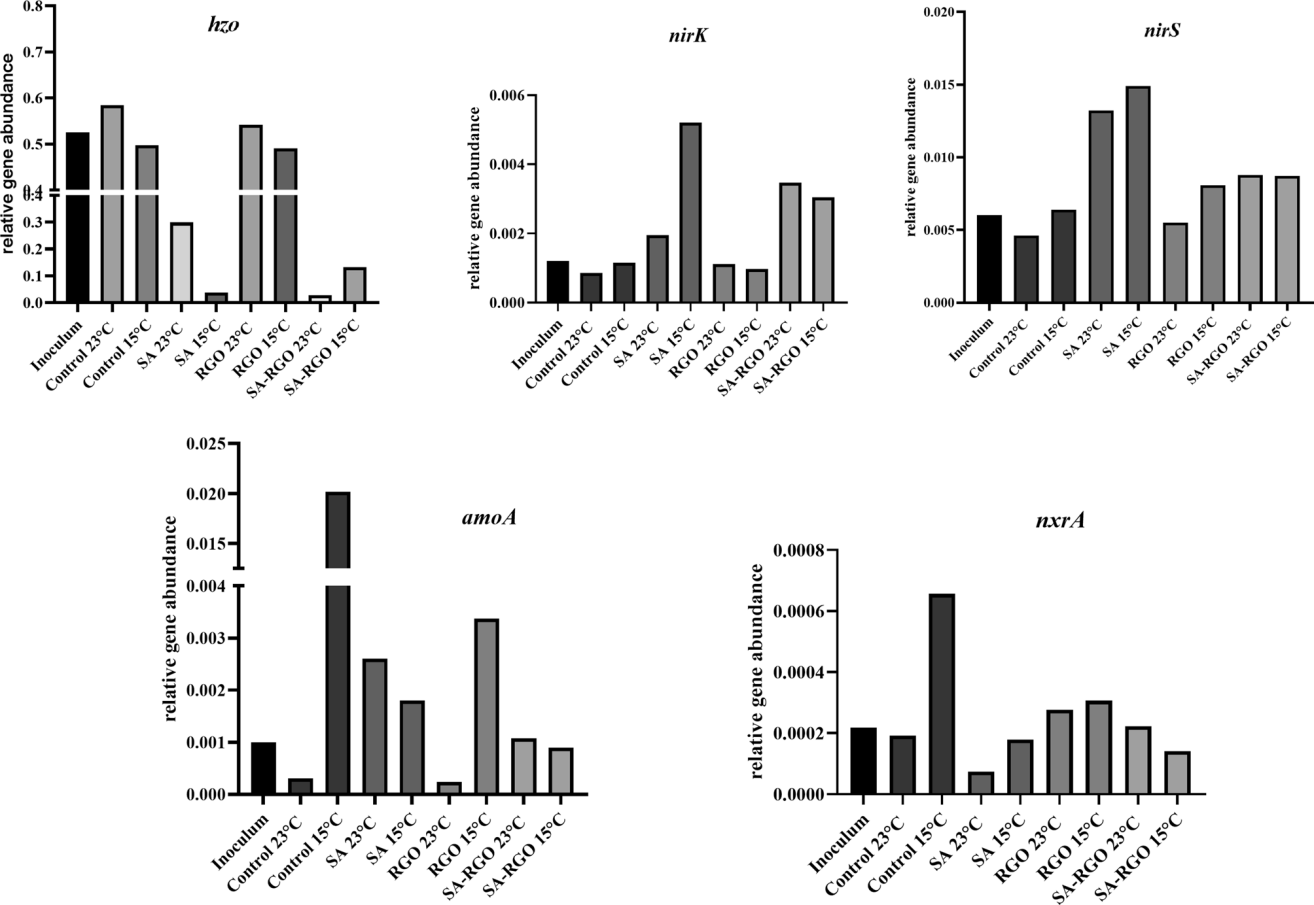
Relative gene abundances calculated for the nxrA, amoA, nirK, nirS, and hzo genes.

**Figure 6:**
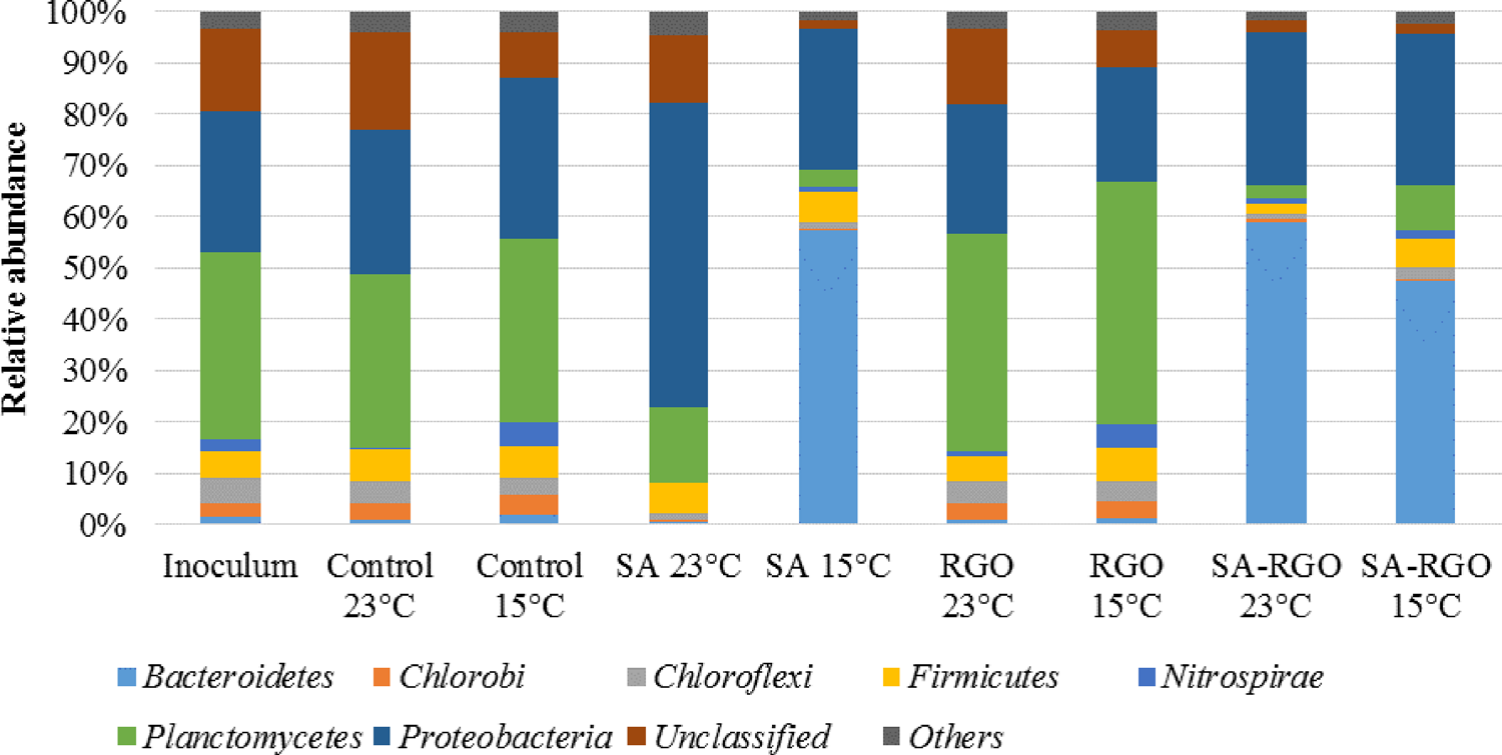
Microbial community composition at a phylum level in the inoculum and investigated SBRs at 23 °C and 15 °C: control reactor, a reactor with RGO addition, a reactor with biomass immobilized in SA, and a reactor with biomass immobilized in RGO.

**Figure 7:**
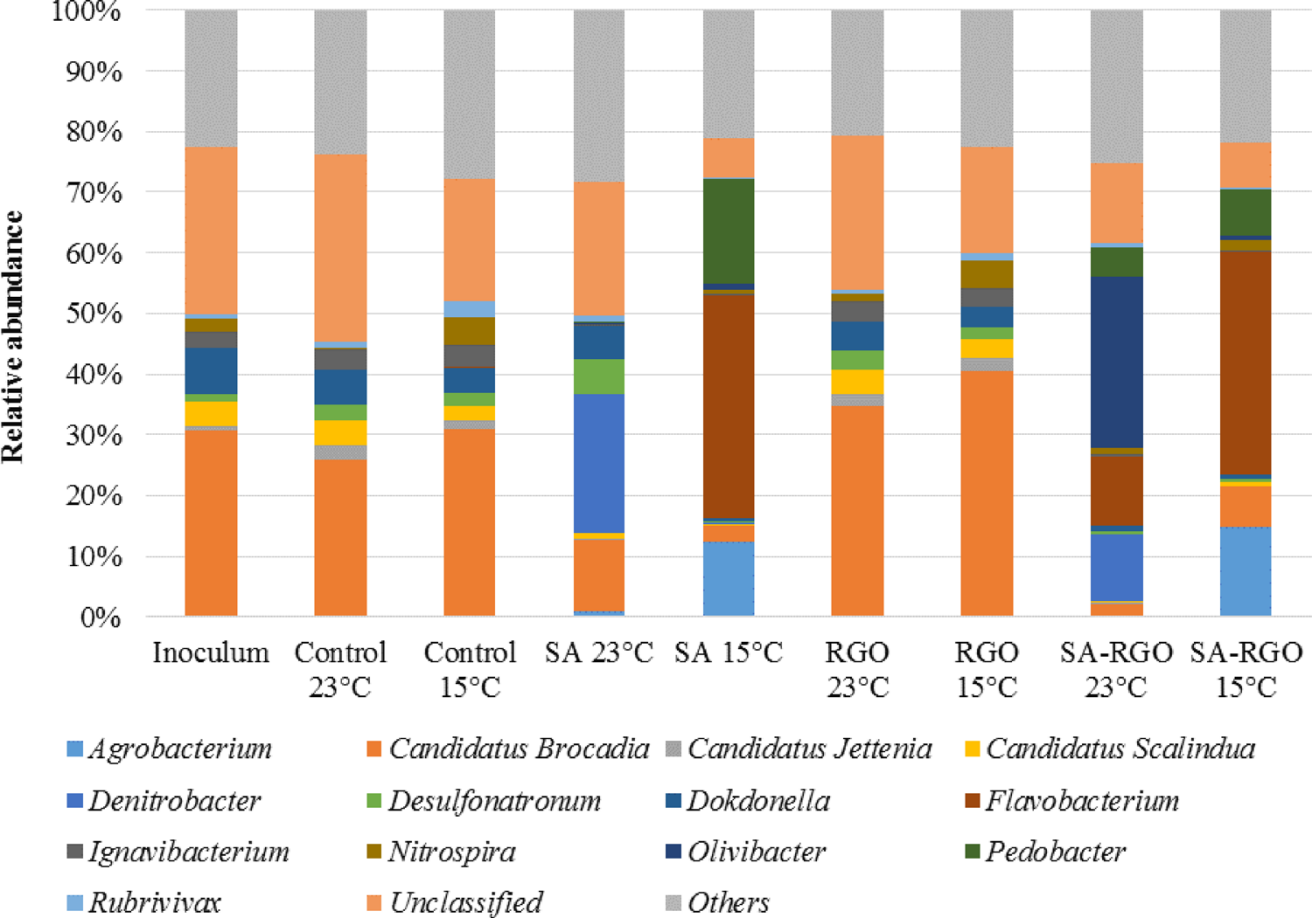
Microbial community composition at genus level in the inoculum and investigated SBRs at 23 °C and 15 °C: control reactor, a reactor with RGO addition, a reactor with biomass immobilized in SA, and a reactor with biomass immobilized in RGO.

Nitrogen removal was monitored in four SBRs according to the type of gel beads used for biomass immobilization and RGO addition at two different temperatures (Figs. 3, 4). The nitrogen removal was more efficient for the immobilized biomass (SA and SA-RGO reactors), both at ambient (23 °C) and at low (15 °C) temperature. The SBRs with immobilized biomass reached a higher nitrogen removal rate (NRR) and nitrogen removal efficiency (NRE). Moreover, the more significant decrease in NRE at 15 °C is observed for the non-immobilized biomass (control and RGO addition) in the comparison to the immobilized samples, this suggests that there are protective properties to the immobilization (Fig. 3a,b). However, the stability of the process carried out in SA and SA-RGO reactors was disturbed, whilst the control and RGO reactors operated in a relatively stable manner, reaching a similar level of NLR [249–338 g N/m^3^*d (control), 213–316 g N/m^3^*d (RGO)] and NRR [131–287 g N/m^3^*d (control), 121–243 g N/m^3^*d (RGO)] (Fig. 4). The deviation in the nitrogen removal efficiency may be associated with the biomass adaptation or microbial community remodelling. At the 11th day of operating the SBRs at 23 °C the theoretical maximum NRE in the anammox process has been exceeded and was equal 91.68–94% (Fig. 3a), this clearly indicates denitrification. During the anammox process, the theoretical ratio of NO_3_-N produced to NH_4_-N removed should be approximately 0.26^52^. A ratio below the theoretical values indicates that nitrogen was removed by denitrification. The NO_3_-N produced to NH_4_-N removed ratio dropped significantly in the SA and SA-RGO reactors after the first week of the experiment at ambient temperatures and stays below this theoretical value, and reaches negative values throughout the entire phase of the process when conducted at low temperatures. The denitrification contribution is also analogous in the molecular biology analyses. The *nirS* and *nirK* genes abundancy increase significantly (p < 0.05) in reactors with biomass immobilized both in SA and SA-RGO beads (Fig. 5). Moreover, the NGS results reveal that in SA and SA-RGO carriers the denitrifiers start to dominate (Figs. 6, 7). The synthetic medium did not contain organic carbon source, suggesting that denitrifiers started to consume the sodium alginate.

### Microbial community structure and key enzymes activity analyses

Nitrogen removal results are summarized and compared with microbial community analysis performed using molecular biology tools. As it has been previously reported, the anammox bacteria, nitrite-oxidizing bacteria (NOB), ammonia-oxidizing bacteria (AOB), and denitrifies are coexisting groups of microorganisms in the anammox biomass^39,40^. In order to get more insight into the microbial community, we determined the relative abundance of *hzo, amoA, nxrA, nirS,* and *nirK* genes (Fig. 5). The investigated SBRs were inoculated with activated sludge performing anammox process. That is why *hzo* gene, specific for anammox bacteria, was the most abundant gene in all the investigated reactors during the experiment. However, NGS and qPCR analysis reveal that representatives of each group of nitrogen removal bacteria were present in the reactors and both the structure of microbial communities inside the beads and relative functional genes abundancy changes significantly, depending on the operational temperature and biomass immobilization/RGO addition. Data from Figures 5, 6, 7 represents microbial structure and key enzymes changes.

The highest *hzo* gene abundance (compared to the control) shows biomass with RGO addition in reactors operated at both 23 °C and 15 °C. However, the differences between the RGO and control reactors were not statistically significant (p = 0.23). The anammox species and their growth rates differ, depending on their ecological niche and environment^9,53^. Whilst the *hzo* gene was the most abundant in the RGO and control reactors, the most abundant phylum was *Planctomycetes* and the most abundant genus was Candidatus *Brocadia* (Figs. 6, 7). It was reported that Candidatus *Brocadia* appeared to have an activity optimum at 20–30 °C. However, high specific anammox activities may be achieved after the adaptation of the mesophilic growing bacteria to low temperatures^54^. Hu et al.^55^ have previously shown that Ca*. Brocadia* is the dominant genus, as corroborated by Winkler et al.^56^ in a reactor operated at 18 °C, and inoculated, similarly as with our studies, with biomass from a 30 °C anammox reactor. This suggests that Ca*. Brocadia* possesses a competitive advantage at low temperatures amongst the anammox microorganisms. The Ca*. Brocadia* abundance increased significantly (p < 0.05) in the reactor with RGO addition and was highest in RGO operated at 15 °C. The positive effect of the RGO addition on cold anammox process was reported by Tomaszewski et al*.*^13,14^. Beside Ca*. Brocadia*, Ca*. Scalindua* and Ca*. Jettenia* were present in the investigated SBRs, however, these represented a much smaller part of the microbial community.

In contradiction to the suspended biomass, a significant drop in the *hzo* gene abundancy is noted in the case of the SA and SA-RGO reactors, while both *nirS* and *nirK* increased significantly (Fig. 5). As was mentioned before, these changes were directly connected to the contribution from denitrification. The correlation coefficient calculated between *hzo* gene abundance and *nirK* and *nirS* are − 0.935 and − 0.772, respectively. The negative correlation suggests that denitrification displaces the anammox process from the reactors with immobilized biomass. The anammox, denitrifies and the AOB and NOB microbial communities can shift and change along with changes in the environmental and imposed operational conditions. The NGS studies suggest that microorganisms are capable of denitrification and start to dominate in SA and SA-RGO reactors. More importantly, bead degradation was noticed at the end of the experiment and together with an increase in the relative amount of *nirS* and *nirK* gene abundance suggests that the denitrifiers started to use the immobilization reagents as an organic carbon source. *Flavobacterium* (phylum *Bacteroidetes*) is present in all of the investigated reactors. About half of the *Flavobacterium* species are able to reduce nitrate to nitrite. Despite this, in the inoculum it was only 0.36% of the total reads represented *Flavobacteriia* during the experiment, the species *Flavobacterium succinicans* presented 5.38, 22.5, 23.25% of total reads in SA-RGO reactor (23 °C) and both the SA and SA-RGO at 15 °C, respectively. The optimal temperature for this species ranges from 2 to less than 37 °C and the bacteria are able to complete nitrate reduction. No data was found about the ability of *Flavobacterium succinicans* against alginate degradation. However, it is worth noting that a few *Flavobacterium* strains give a positive response for alginates, such as *Flavobacterium johnsoniae* presented in investigated sludge in SBRs with sodium alginate^57^. One of the most intriguing *Flavobacterium* species presented in SA and SA-RGO reactors operated in 15 °C is a facultative anaerobe named *Flavobacterium denitrificans*, which grows by carrying out complete denitrification in the mineral medium, producing N_2_O as a transient intermediate during the reduction of nitrate to nitrite and using O_2_, NO_3_^−^, and NO_2_^−^ as electron acceptors^58,59^. Another genus with denitrifying ability, presented in SA and SA-RGO reactors operated at 23 °C is *Denitrobacter*. This genus represents 22.92 and 11.10% of the total reads, for the SA and SA-RGO reactors, respectively.

The members of the nitrifying community were presented, as well as denitrifires and anammox bacteria in investigated reactors. Besides the anammox bacteria, genes from the AOB fraction were all relatively high in abundance amongst the nitrogen removal community, especially in the control reactor at a low temperature. The possible activity of the AOB bacteria in the anammox biomass and the presence of nitrification was also confirmed in our previous work^39,40^. For each investigated condition, *amoA* gene, specific to AOB, was more abundant than the *nxrA* gene*,* the one characteristic of NOB. The AOB is often the dominant group over the NOB in a microbial community in WWTP systems and when nitrification is present^56,60,61^. However, the *nxrA* gene and microorganisms related to nitrite oxidation such as *Nitrospira* were also detected in investigated SBRs. Recent studies indicate that *Nitrospira* is one of the most dominant NOB species in wastewater systems^62–65^. Even if the inoculum was taken from anammox reactor, *Nitrospira* only represented 2.27% of total reads and has double the abundance in the control and RGO reactors at 15 °C (4.53% of total reads, both in the control and RGO). Also, the *nxrA* gene was more abundant in the control and RGO reactors, especially at a low temperature, and significantly increased from 1.92 × 10^–4^ at 23 °C to 6.56 × 10^–4^ at 15 °C in the control reactor. It follows, that nitrifying bacteria prefer temperatures similar to the main sewage treatment plant^66^.

A specific species of *Nitrospira* can perform both nitrition and nitration. The existence of a complete ammonia oxidizer (comammox) was reported recently^67^. Key functional genes characteristic for comammox *Nitrospira* have been detected in diverse aquatic and terrestrial environments as well as in new metagenomes from wastewater treatment plants^65,68^. Due to favourable conditions in the investigated the SBRs, it is highly probable that in the control or RGO reactors the specific comammox microorganisms are present.

## Conclusions

RGO addition improved the properties of SA gel beads by suppressing the water-related variables. Nitrogen removal was more effective in the case of immobilized biomass, compared to non-immobilized, particularly at a temperature close to the mainstream of the municipal WWTP. However, molecular biology analysis revealed that the microbial communities' and their composition changes significantly, depending on the operational temperature and biomass immobilization or RGO addition. This indicates that whole biomass entrapment in the gel beads did not support the anammox bacteria grow, instead of that, affected the microbial community structure remodeling and increased the denitrification contribution.
